# Maintaining the optimum level of immunosuppressive agents minimizes risk of liver allograft fibrosis during pediatric liver transplantation

**DOI:** 10.1097/JS9.0000000000000759

**Published:** 2024-05-10

**Authors:** Mebrahtu G. Tedla, Mebrihit M. Kahsay, Mebrahtu G. Kidanu

**Affiliations:** a Department of Pediatrics, School of Medicine, University of Missouri-Columbia, Columbia, Missouri, USA; b School of Medicine, College of Health Sciences, Addis Ababa University, Addis Ababa; c Department of Pediatrics, School of Medicine, College of Health Sciences, Axum University, Axum, Ethiopia

*Dear Editor*,

A very recent study by Jiang *et al*.^1^ conducted a retrospective cohort study to examine the effect of immunosuppressive level on liver allograft fibrosis (LAF) after liver transplantation in children and showed even in children with adequate liver function, a histologically proven mild to moderate LAF was very common, and its severity tended to worsen over time. In their finding, they also reported following liver transplantation, the immunosuppressive regimen should be modified over time based on the clinical condition of the patients. The excessive buildup of extracellular matrix proteins, such as collagen, within the transplanted liver is known as allograft fibrosis. The activation of portal fibroblasts and hepatic stellate cells in response to chronic damage is the main event. Progressive LAF invariably causes graft failure and loss when left untreated^2^. LAF is one of the key elements influencing children’s long-term graft survival following liver transplantation in children^1^. Pediatric liver transplantation (PLT) is a lifesaving surgical intervention to manage acute, chronic, and end stages of liver disease, and it is considered the best treatment option when all types of medical treatments fail to cure liver diseases^3,4^. Studies showed that the survival rate of children who had liver transplantation is about 90% 1 year after the treatment and 77% in 5 years^5^. Effective and successful graft survival following liver transplantation can be achieved using appropriate immunosuppression drugs. LAF was significantly linked with transplant-related variables such as long cold ischemia times, young liver transplant ages, large donor-to-recipient age ratios, and partial graft usage^6,7^. According to Scheenstra *et al*.^6^ at 1, 3, 5, and 10 years following liver transplantation, respectively, the prevalence of LAF increased from 34 to 48, 65, and 69% among children. Additionally, it has been shown that LAF is becoming more common and severe with time. Only 30% of patients are anticipated to have normal histology 10 years following liver transplantation^8^.

Managing immunosuppression regimens in children is complex and challenging compared with adult patients. However, the type and dose of immunosuppressive drugs and medical therapy after transplantation have led to dramatically improved outcomes in children with acute and chronic end-stage liver disease^9^. During PLT, better graft survival and long-term outcomes are also attributed to choices in the immunosuppression regimens^10^. As a result, acute and cellular rejection, graft loss and dysfunction can be prevented^11^. To reduce allograft rejection after liver transplantation, immunosuppressants are widely employed. Immunosuppressants may encourage cell death, which triggers an inflammatory response that encourages fibrosis, according to experimental findings from non-liver cells, raising worries that a similar impact may occur within the liver^12^.

Following the transplantation, evaluation of the liver allograft in long-term survivors is also very crucial for an effective outcome. Unlike in adults, in which recurrence of primary diseases contributes to draft failure and dysfunction, in children, liver fibrosis and idiopathic hepatitis are the main factors that cause late graft dysfunction^3^. It’s important to strike a balance between preventing organ rejection and reducing potential adverse effects when it comes to immunosuppressive medications and LAF in children after liver transplantation. The dosage of immunosuppressive medications will vary depending on a number of variables, including the particular medication used, the child’s age, weight, and general state of health. Immunosuppressive therapy’s main objective is to reduce the recipient’s immunological response in order to stop the transplanted liver from being rejected. A LAF is one consequence that can result from prolonged and severe immunosuppression. The extent and duration of immunosuppressive medication is one of many variables that affect fibrosis development.

In conclusion, during PLT, it is important to carefully monitor immunosuppressive medicine levels in the child’s blood to reduce the danger of LAF. As a result, they can modify the dosage as necessary to keep the drug levels within the therapeutic range and prevent toxicity. To evaluate the condition of the liver and identify any early fibrosis indications, routine medical exams and liver function tests should be carried out. To assess the degree of fibrosis, additional diagnostic methods such as liver biopsies or non-invasive testing may also be performed. Therefore, managing immunosuppression and lowering the risk of LAF in children receiving liver transplantation requires adjusting the medicine dosage based on the child’s unique reaction while taking into account characteristics including age, weight, and liver function.

## Ethical approval

No human or animal patients have been involved in this study. Thus, ethical approval was not needed.

## Consent

This study didn’t employ any patients, and as a result, there is nothing to declare.

## Sources of funding

No funding was received for this work.

## Author contribution

M.G.T.: conceived the idea and drafted the paper; M.G.K. and M.M.K.: gathered relevant data and edited the paper. All authors read and approved the final version.

## Conflicts of interest disclosure

The authors declare no conflict of interest.

## Research registration unique identifying number (UIN)

1. Name of the registry: not applicable.
2. Unique identifying number or registration ID: not applicable.
3. Hyperlink to your specific registration (must be publicly accessible and will be checked): not applicable.

## Guarantor

Mebrahtu G. Tedla, Child Health Research Institute, School of Medicine, University of Missouri-Columbia, Columbia, Missouri 65211, USA; E-mail: mtmpx@health.missouri.com. M.G.T. takes full responsibility for the validity and accuracy of the finding.

## Data availability statement

No primary data has been collected for this particular study.

## Provenance and peer review

Not commissioned.
